# Caregiving in rural areas: A qualitative study of challenges and resilience

**DOI:** 10.1371/journal.pone.0325536

**Published:** 2025-06-06

**Authors:** Hyojin Choi, Christine Vatovec, Maija Reblin

**Affiliations:** 1 Department of Family Medicine, University of Vermont, Burlington, Vermont, United States of America; 2 College of Nursing and Health Sciences, University of Vermont, Burlington, Vermont, United States of America; University of Minnesota, UNITED STATES OF AMERICA

## Abstract

**Background and objectives:**

Informal caregivers are critical to providing support to adults with functional limitations or disease in rural areas. There is a need to understand factors that impact rural caregiving to build equitable health services, yet there is a lack of research that weighs both strengths and challenges. Our goal is to examine these factors as identified by rural caregivers in qualitative interviews.

**Research design and methods:**

A purposive sample of rural-dwelling caregivers (n = 18) was recruited from a state-wide registry in a predominantly rural state. Semi-structured interviews were conducted by phone and interview transcripts were qualitatively analyzed.

**Results:**

We identified areas of strengths and resilience among rural caregivers, including informal support from friends and neighbors, which often bridged gaps in formal services. Rural caregivers also identified barriers to service use, such as long distance, travel and a lack of awareness within the healthcare system.

**Discussion and implications:**

These challenges increase caregivers’ needs for tailored family centered care coordination and highlight the necessity of providing diverse care delivery options. Our findings highlight that community-based efforts and policy should address barriers and rural disadvantage, while also fostering and empowering the resilience of caregivers in their rural home environment.

## Introduction

Populations living in rural areas face greater health risks than those in urban areas [1]. This is due to demographic factors such as a significant proportion of older adults [2] as well as socially-structured disadvantages, such as limited transportation and low accessibility of healthcare services. As such, family caregivers– defined as a those who provide any support or assistant for their family members or friends with functional limitations or disease – play a critical role in rural health care. Rural residents are more likely to receive unpaid care from family members or friends and less likely to receive formal, paid care [3].

**Fig 1:**
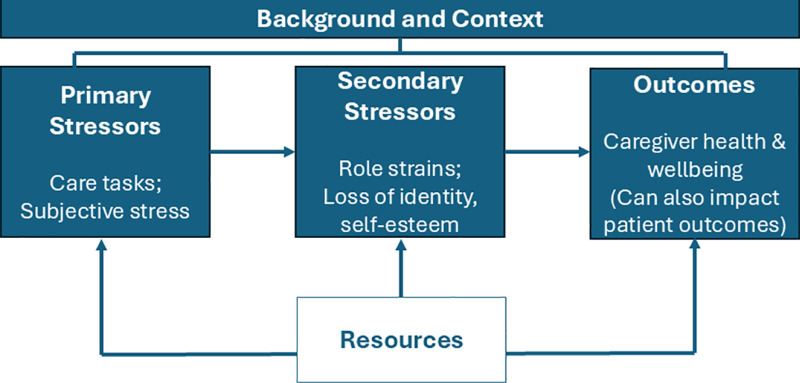
Adapted stress process model, Pearlin et al.

Although providing care can be meaningful [4], many caregivers experience challenges to their own health and well-being because of these added responsibilities [5]. As outlined by the Stress Process Model [6] and shown in Fig 1, primary stressors related to care tasks, along with secondary stressors relevant to role strain can lead to poor caregiver outcomes, such as worse caregiver health and well-being, which can spill over to negatively impact patient outcomes. However, access to resources can buffer the impact of stress and protect against these negative outcomes. Resources can come in the form of direct assistance, such as help from healthcare or other formal services or others helping to manage caregiving or other stressful tasks, such as work or family conflict. Resources can also come in the form of psychological resilience, such as the ability to engage in adaptive coping strategies, manage difficult emotions, and reframe a difficult experience as a growth opportunity.

Caregivers in rural areas may face disadvantages that can impact the health and well-being of family caregivers due to fewer formal services and availability of support. For example, compared to caregivers in suburban or urban areas, those living in rural areas face greater challenges in finding affordable services and have fewer interactions with healthcare providers [7], which can make caregiving more difficult. Despite structural challenges, rural residents and their family caregivers often report strengths and assets living in rural areas. Twenty percent of Americans live in rural areas [8], and this group has the highest satisfaction with where they live [9]. In a recent systematic review paper [10], strong personal networks in rural communities, and sense of connection and safety were identified as strengths among family caregivers in rural settings. Living in a rural community may also confer certain benefits that remain unexplored in academic literature. There has been a lack of high-quality research that explains mechanisms around benefits to living in rural areas [11].

There is a need to address health disparities in rural areas, including those related to caregiving. Most of the disadvantages associated with living in rural areas are not inherent to these places. In fact, living in rural areas was protective for health until the 1980s [12], suggesting that changing public health policy and infrastructure as well as policies that impact poverty may be critical factors. As such, it may be possible to reverse these disparities and restore equity for those living in rural areas. Although barriers need to be identified and addressed, it is also important for policies to recognize and capitalize on strengths that may exist in rural areas to effect lasting change [13].

The purpose of our study is to examine the factors that rural-dwelling caregivers themselves identify as relevant to caregiving in rural location. The findings of this study will contribute to identifying strengths to capitalize upon and responding to health disparities to advance rural health equity.

## Research design and methods

We take a narrative research approach within a constructivist research paradigm. As such, we interpret the lived experience of rural-dwelling caregivers using their own words and their descriptions of meaning. The authorship team includes trained qualitative researchers who also have lived in rural areas, which may influence our assumptions and interpretations. Although there was no prior relationship between the research team and participants, there is some familiarity with the health care institutions and areas discussed by caregivers in interviews.

### Data collection

Semi-structured interviews were conducted between February 19th and March 29th, 2024. Research was conducted in a state with a highly rural state, with a high proportion of older adults [8]. Interviews explored the experiences and perspectives of rural caregivers on their needs, interest, barriers and facilitators around formal service use. A study team member trained in qualitative research conducted in-depth interviews via phone. Interviews took approximately 30–60 minutes, audio-recorded, and transcribed. All participants were provided an information sheet summarizing the research study prior to enrollment. This was discussed with research staff and verbal informed consent was obtained for all participants. All files were stored on secure servers. No identifying information is presented in this manuscript. This study was performed in line with the principles of the Declaration of Helsinki. All research activities were approved as exempt by the University of Vermont IRB (STUDY00002909) prior to study implementation. Verbal informed consent was obtained from all individual participants included in the study. A waiver of documentation of this consent was approved by the IRB. The purpose-driven semi-structured interview guide is available in S1 File.

### Participants

To identify caregivers in rural areas, data of the Vermont Caregiver Registry was utilized. Adult participants who self-identified as a caregiver to another adult were recruited to the registry through clinical and community settings. Registry participants completed basic demographic information and indicated willingness to participate in research studies. From registry participants interested in research, we purposively sampled for rural-dwelling caregivers Rurality was using the US Office of Management and Budget definition of living in a city or town with population less than 10,000 [14].

### Analysis

Descriptive analysis was conducted on demographic characteristics of the sample. A qualitative content analysis was conducted on interview data. The interview data was anonymized, identified by participant code number. Participant numbers are listed after each quote presented below. First, three study team members reviewed transcripts to familiarize themselves with interview content. Then, the last author identified areas with discussion relevant to the participant’s rural location or local context. Responses were inductively categorized into themes within broader concepts of benefits and barriers of caregiving in a rural area. These findings were discussed among the study team to ensure credibility, and coder notes were made throughout the coding process. Any disagreements were resolved by consensus.

## Results

### Demographics

Thirty-six caregivers were approached and 18 completed an interview. Demographics are presented in Table 1.

**Table 1:** Demographic characteristics of the sample (n = 18).

	Mean (S.D.)		
Age	59.7 (12.7)		
	**N (%)**		**N (%)**
Gender		Relationship to primary care recipient	
Female	16 (89)	Spouse or partner	9 (50)
Male	2 (11)	Parent (or parent in-law)	5 (28)
		Others (child, sibling, friend)	4 (22)
Race		Living with care recipient	
White	18 (100)	Yes	11 (61)
Ethnicity		No	7 (39)
Non-Hispanic or Latino	16 (89)	Number of care recipients	
Unknown/Not reported	2 (11)	One	13 (72)
Education		Two	3 (17)
Some college	4 (22)	Three or more	2 (11)
Bachelors degree	5 (28)	Care recipient’s health conditions^a^	
Graduate or professional degree	9 (50)	Cancer	6 (33)
Employment		Dementia	6 (33)
Employed full-time or part-time	8 (44.5)	Mobility issues	5 (28)
Retired	8 (44.5)	Mental health	4 (22)
Other	2 (11)	Old age/Frailty	3 (17)
Financial situation		Diabetes	3 (17)
Not very good	3 (17)	Heart Disease	3 (17)
Comfortable	14 (78)	Stroke	3 (17)
More than adequate	1 (6)	Other	14 (78)
Caregiver self-reported health		Caregiving duration	
Fair	2 (11)	Less than one year	5 (28)
Good	5 (28)	Two years	4 (22)
Very good	4 (22)	Three or more years	9 (50)
Excellent	1 (6)		

^a^Care recipient’s health conditions are not mutually exclusive

### Qualitative findings

#### Rural resources for resilience.

Although all caregivers identified barriers to engaging in their role in rural areas, most mentioned that they had intentionally chosen where to live, despite any tradeoffs:

*Our choice is to live [in a rural area] in the winter. It’s slippery and your chances of falling are greater, but that’s the pleasure of living [here]. (74)*

Some caregivers mentioned a sense of place as an important justification for living where they did:

*I live in the middle of nowhere…obviously in a beautiful little town… on a gorgeous lake. (108)*

Similarly, local natural spaces were mentioned as important sources of respite.

*I do enjoy walking, and I do enjoy spending time by the water… I love to drive over [to the lake] and… just look out at the water. (131)*

Taken together, the way caregivers discussed their rural hometowns suggested that they drew comfort and pleasure in place, and these rural areas offered a source of strength and respite. In contrast, urban areas were sometimes discussed as adding more stress with crowds and traffic, and caregivers preferred the familiarity of home.

Although a lack of high-quality resources and a desire for independence were identified as barriers, as described below, a sense of community and personal relationships were identified as a common benefit to living in rural areas.

*[After a storm downed trees on the caregiver’s property, a community member] said, “Well, OK, then I’ll go over and see if I can help them’… People who live in a city don’t see that as much… I never had seen anything like the people out here until I moved out into the country…They figure if you’re dumb enough to live in the boonies, help others. (118)*

One way people could maintain independence while benefiting from neighborly support was to trade tasks and understand that there was an expectation to help each other in some way:

*We have two young families next to us and my husband, he can’t walk. But he can drive. So, we got sort of a hand-me-down riding mower, and he cuts everybody’s grass. And everybody sort of does stuff for each other…sometimes it involves like oh, we have these vegetables that we grew, we’re bringing them to you and other times it’s will you watch my child while I’m on a phone call. (23)*

Sometimes these critical relationships extended specifically into a healthcare context. One caregiver mentioned that living in a small town meant her mother had developed a personal relationship with her doctor.

*This doctor actually listens and understands what stage of life mom is in…I think most people don’t have that anymore. So, I feel very fortunate that we have a doctor in our town who mom has seen for a long time. (60)*

These personal relationships were critical resources that helped caregivers living in rural areas cope with stressors related to caregiving as well as secondary stressors, such as daily hassles.

#### Challenges to accessing services in a rural area.

One main theme identified as a barrier to caregiving in a rural area was challenges in accessing high-quality services locally.

*Where is there a whole lot of choice? …we have very few resources. We do technically have a hospital. It has very few providers in it because, particularly since COVID decimated our area… all of the resources went away. (108)*

*We’ve tried to get PT* [physical therapy] *to come in to help him, which that in itself has been a major struggle because the PT person that they send is pretty much useless. We’ve complained about it and they just say, OK, the other services are terminated and that’s it. They don’t provide somebody else. They say, well, there’s nobody else covering that area. (103)*

While some services were available online, this was not seen as a viable or desirable option.

*One struggle I am finding is… there doesn’t seem to be [a support group] in person. There’s online… but I don’t really want to do online. I want to know someone in my community… down the street is dealing with the same thing and maybe we can get together. (134)*

Generally, caregivers lamented the lack of additional resources but had varying degrees of acceptance. Some seemed to accept that fewer resources and connections were part of the rural experience, though more reported that more should be done to extend services out to rural areas.

This lack of access also somewhat extended to informal support networks. Many had deep social connections with other residents nearby. However, for others, especially those who did not grow up in the area or those whose families had moved, family and friends were seen as supportive, but distance created challenges to accessing that support.

*My daughter’s been wonderful. She calls all the time, she video chats with the grandkids, and that has been a big support… A lot of my friends move next to their grandkids, and I said that makes a lot of sense because, you know, it would be great to be able to say, hey, can you come over for an afternoon and just watch Dad while I go do something? (135)*

#### Travel interacts with other barriers.

Because of the lack of local access, most caregivers traveled to services, which was frequently identified as another barrier to caregiving in rural areas. However, some mentioned that they were just used to the difficult conditions, so travel was less of a concern for them.

*I kind of brush it off like that was an inconvenience to drive 6 hours [to an appointment] but I was comfortable doing it…I grew up… driving in the snow. And I drive others in the snow to appointments and things like that. I’ve got a great little 4-wheel drive vehicle with studded snow tires and I don’t care. I’ll drive in any condition (60)*

Commonly, caregivers mentioned that the travel time to appointments in more urban areas added time to their usual responsibilities:

*There’s some days where it’s like a couple appointments a week… It gets tricky trying to get it all in…Especially because the doctors are up in [the city], we live in [a smaller town], so you add that additional hour each way. It’s a lot. (Caregiver 22)*

This was often compounded with caregivers’ own health needs and medical appointments, which could not be addressed in the same location:

*I usually have to have CT scans and different tests before I get to my pulmonary appointment... We live 3 1/2 hours away [from the care recipient’s doctor], but my pulmonary is an hour and a half the other direction. (135)*

Similarly, older caregivers often struggled with transportation over long distances in poor conditions:

*We don’t have anything in our town or close by that he can go to. It’s always a trip… When something happens and it’s blowing snow at night, or the roads are icy… I don’t drive in the dark if I don’t have to. Both of us are older, and so I said I’m going to call the ambulance. I’m afraid to drive you in, you know? (118)*

Caregivers sometimes felt that providers were not sensitive to the difficulty distance posed for them in providing care:

*They don’t necessarily tell you when she’s going to be discharged… I was on my way to get her [from several hours away] and she called and said they’re not discharging me today, so I had to stop and turn around and come home. I’m not knocking health care providers because certainly they do a good job, but they do not care about caregivers. That is the least of their concerns. (26)*

#### A need for privacy and independence.

Finally, some caregivers identified a desire for privacy or independence as a barrier to engaging in more support that would make caregiving in rural areas easier. This included privacy around the care recipient’s body:

*Not everybody is comfortable dealing with bodies… I think the challenge for other caregivers--particularly if it’s not a family member; if it’s a family member, you can [engage in toileting or other intimate care.] But if it’s not a family member, that’s a big deal. (108)*

But also included their own personal space.

*It’s not like we need somebody to come once a week and help them take a shower and wash… It’s the kind of thing that any moment he could start having pain. So, whoever was going to help me or give me respite would have to just stay here, and we don’t want somebody who live in here with us, just in case [they were needed]. (118)*

## Discussion and implications

Extensive research has demonstrated that people in rural areas are more likely to experience health disparities [15,16]. This impacts those in need of care, as well as the family members and friends who provide care. Therefore, it is important to recognize the broader context in which people in rural areas live, which offers familiarity, areas of respite, and critical networks of community support. There is a need to develop rural healthcare delivery systems that recognize and acknowledge the strengths of rural areas, while addressing the logistical challenges residents face in providing care.

Caregivers in our sample were mostly long-time residents of the rural area in which they were providing care, rather than newcomers without existing social networks. The existence of strong social networks and familiarity of the available resources and environment of “home” can be important resources to help cope with caregiving responsibilities. In contrast, although moving to an urban area may come with better access to more services, the need to learn a new environment and the lack of a developed social network may add additional stress. Caregivers in rural areas in our sample identified several features of their environment that led to resilience, or the ability to bounce back or experience positive outcomes in the face of adversity [17]. These included particular mindsets common to those living in rural areas as well as specific resources available to them. For example, many participants noted acceptance of potential stressors or barriers to caregiving, such as long transportation times or lack of services, and noted that sometimes this was simply a tradeoff for living where they did. This cognitive reframing and acceptance are important aspects to coping that can help build resilience and reduce negative well-being in caregivers.

The specific resources noted by our participants included both nature and beauty and a sense of community. In different ways, these offer sources of respite and coping. Rural residents also often have easier access to nature than those living in urban areas and nature exposure has shown important health benefits [18], including building resilience and enhancing restoration [19]. Being outdoors can provide a respite emotionally, allowing caregivers to recenter and take a moment of respite. Further, many choose to engage in physical activity in nature, which can further enhance coping and resilience. Research suggests that living in an environment that supports physical and social activity is linked to better outcomes for older adults [20]. Preserving and acknowledging these resources and their importance in coping with stressors such as caregiving, is critical to maintaining the health of caregivers in rural areas.

Similarly, existing strong networks of community support were identified as a resource in our study, in line with other research [21] and our conceptual model. This informal support can be an important backstop to gaps in formal services common in rural areas. Social support can offer critical resources to share caregiving tasks, deal with other stressors such as household management, or simply gain a place to vent, get advice or empathy, and receive validation. However, one area of tension in our findings is the strength of community aligned with the desire for privacy and independence. In other work, desire for privacy was associated with healthcare avoidance [22] and many caregivers avoid asking for help because of a desire to maintain control and to avoid burdening others [23]. There may be a hierarchy of support-seeking based on role and task. For example, personal toileting may be reserved for family only, whereas home or yard maintenance may be more acceptable for community help. Understanding these boundaries and distinctions can help inform how friends and neighbors can best extend offers of support to help bridge gaps in tangible formal support that can be difficult to deliver in person.

Although rural caregivers identified strengths gained from living in rural areas, barriers were also identified. One critical barrier frequently identified by caregivers was distance and travel, although some participants were accustomed to transportation issues. Needing to travel to take a patient to clinical treatment can increase risky health behaviors (e.g., relying on convenience foods), disrupt sleep quality by staying in an unfamiliar environment, and reduce social contact with friends [24], which contribute to worsening health outcomes of family caregivers. Therefore, there is a need for developing interventions aimed at caregivers that acknowledge the importance of prioritizing their own health and support healthy behaviors—and account for the geographic location of caregivers to ensure that both urban and rural people can thrive in their caregiving roles. Some healthcare systems, especially in the wake of the COVID-19 pandemic, have expanded telehealth offerings or outpatient treatment options [25,26]. Although this can be beneficial to close some gaps, some caregivers in our research and others have been shown to have negative feelings about telehealth and challenges have been identified in learning and using telehealth technology [27]. Services like mobile units may be more acceptable in rural areas, with telehealth used only as a bridge between in-person visits, instead of as a replacement.

Clear, timely communication between patients, caregivers, and healthcare providers is a critical component of high quality care coordination [28]. In our study, caregivers noted a lack of awareness within the healthcare system of the barriers they faced in receiving or coordinating care, and this is also reflected in other work that identifies rural/urban differences in patient perception of care coordination related to communication challenges [29]. Better communication between caregivers and providers has been shown to improve caregiver preparation and patient outcomes [30]; the impact of strong relationships with healthcare providers was also noted as beneficial to caregivers in our sample. Our findings suggest that a benefit of living in rural areas is the potential for patients to develop long-term relationships with healthcare providers, and this can help provide a foundation for building better communication and coordinated care, creating environments where they feel comfortable asking questions, and feel respected and supported [31]. This is a resource that could be capitalized upon and suggests preserving rural clinics and supporting more family medicine or primary care clinics.

## Limitations

Our study has several limitations. First, our sample was relatively small and from one area of the U.S. with low racial/ethnic diversity. Previous studies highlight that rural health disparities intersect with racial and ethnicity [15], so future research should consider more diverse rural populations. In addition, our sample comprised caregivers who had opted-in to research. Some research suggests that rural residents may be more hesitant to participate in research which may limit generalizability; however, this may be due to fewer opportunities for research [32].

## Conclusion

Rural disadvantage impacts not only individuals who need care but also those who contribute to their care. Caregivers, especially in rural areas, are at the forefront in providing appropriate support to care recipients. It is, therefore, critical to understand their lived experience and support their health and well-being and consider both the socially structured factors influencing their choice and the strengths or assets they have [16]. Future research should focus on addressing barriers, for example by using technology or existing resources to extend formal services to rural areas and improving family centered care coordination, while preserving the key strengths caregivers find in their rural home environment.

## Supporting information

S1 File Caregiver semi-structured interview guide. (DOCX)

S2 File Standards for Reporting Qualitative Research (SRQR). (DOCX)

S3 File Correspondence_for_STUDY00002909. (DOCX)

S1 Checklist PLOSOne_Human_Subjects_Research_Checklist. (DOCX)
