# Supplementary material for: Caregiving in rural areas: A qualitative study of challenges and resilience
Source: PLoS One. 2025 Jun 6;20(6):e0325536. doi: 10.1371/journal.pone.0325536 (PMC12143517; doi:10.1371/journal.pone.0325536)
Supplement: S1 File — (DOCX) [file pone.0325536.s001.docx]

**S1.** Caregiver semi-structured interview guide

1. First, it would be great to hear a little background about your caregiving experience.
2. Can you tell me how you protect your own health and well-being as a caregiver, if you do anything? What are some of the challenges you face in taking care of yourself while also caring for someone else?
3. Can you tell me where you get support or what resources you use for caregiving, if any? What has been helpful and what hasn’t? Why?
4. Are there services that are not yet available but would be helpful to you as a caregiver that you would like to see in your area? Why or why not? What would make it easier to access any services that you might be interested in?
5. Are there services that would help you take care of your own health and well-being that you haven’t used or would like to see in your area? Why or why not? What would make it easier to access any services that you might be interested in?
6. Have you, or the person you care for, experienced any problems with some of the recent natural disasters in our area (e.g. flooding, smoke, heat), or do you have any concerns about weather or climate related to being a caregiver?
7. Is there anything else you think we should know or should be asking about with regard to being a caregiver?
