# Supplementary material for: Caregiving in rural areas: A qualitative study of challenges and resilience
Source: PLoS One. 2025 Jun 6;20(6):e0325536. doi: 10.1371/journal.pone.0325536 (PMC12143517; doi:10.1371/journal.pone.0325536)
Supplement: S2 File — (DOCX) [file pone.0325536.s002.docx]

**S2**. Standards for Reporting Qualitative Research (SRQR)

| **No.** | **Topic** | **Page/line no(s)** |
| --- | --- | --- |
| **Title and abstract** | | |
| S1 | Title | 1 |
| S2 | Abstract | 2 |
| **Introduction** | | |
| S3 | Problem formulation | 4-5 |
| S4 | Purpose or research question | 5 |
| **Methods** | | |
| S5 | Qualitative approach and research paradigm | 5 |
| S6 | Researcher characteristics and reflexibility | 5 |
| S7 | Context | 5 |
| S8 | Sampling strategy | 6 |
| S9 | Ethical issues pertaining to human subjects | 6 |
| S10 | Data collection methods | 5-6 |
| S11 | Data collection instruments and technologies | 5-6, S1 |
| S12 | Units of study | Table 1 |
| S13 | Data processing | 5-6 |
| S14 | Data analysis | 6 |
| S15 | Techniques to enhance trustworthiness | 6 |
| **Results/findings** | | |
| S16 | Synthesis and interpretation | 7-12 |
| S17 | Links to empirical data | 7-12 |
| **Discussion** |  |  |
| S18 | Integration with prior work, implications, transferability, and contribution(s) to the field | 12-15 |
| S19 | Limitations | 15 |
| **Others** | | |
| S20 | Conflicts of interest | 1 |
| S21 | Funding | 1 |
