# Supplementary material for: Caregiving in rural areas: A qualitative study of challenges and resilience
Source: PLoS One. 2025 Jun 6;20(6):e0325536. doi: 10.1371/journal.pone.0325536 (PMC12143517; doi:10.1371/journal.pone.0325536)
Supplement: S3 File — (DOCX) [file pone.0325536.s003.docx]

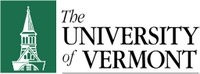


Committees on Human Subjects Serving the University of Vermont and the UVM Health Network

RESEARCH PROTECTIONS OFFICE

213 Waterman Building

85 South Prospect St

Burlington, Vermont 05405

Email – [irb@uvm.edu](mailto:irb@uvm.edu)

[Human Subjects Research](https://www.uvm.edu/rpo/human-subjects-research)

**Exemption Certification - Initial**

| To: | Maija Reblin |  |
| --- | --- | --- |
| From: | Diana Naser, IRB Regulatory Analyst | 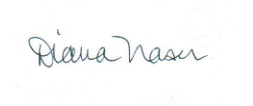 |
| Approved Date: | January 11, 2024 |  |
| Study#: | CHRBSS (Behavioral): STUDY00002909 |  |
| Study Title: | Caring for Caregivers: Assessing Health and Wellness Needs Among Rural Caregivers |  |
| Sponsor: | Internal Funding |  |
| Finalized Documents: | Exempt protocol ; Reblin Caregivers Research Information Sheet SUBMITv2.pdf ; Reblin Rural Caregivers Questionnaire SUBMIT.pdf ; Research Invitation Email Phone SUBMIT.pdf |  |

The study referenced above was reviewed by the Chair of the IRB (or an authorized designee) using the exempt procedures set forth under 45 CFR 46.104. While the project is exempt from IRB review, it is required that researchers follow all human subject protection regulations and notify the IRB of any problems that arise during the conduct of the project.

**Exemption Category: (2)(ii) Tests, surveys, interviews, or observation (low risk)**

(2) Research that only includes interactions involving educational tests (cognitive, diagnostic, aptitude, achievement), survey procedures, interview procedures, or observation of public behavior (including visual or auditory recording) if at least one of the following criteria is met: (ii) Any disclosure of the human subjects’ responses outside the research would not reasonably place the subjects at risk of criminal or civil liability or be damaging to the subjects’ financial standing, employability, educational advancement, or reputation

Consent/HIPAA/Waiver Determinations:

•Waiver of Documentation of Consent under 46.117(c)(1)

This determination applies only to the activities described in this IRB submission and will no longer apply should any changes be made. If changes are necessary, please submit a modification for consideration of a continued exemption.
